# Perception of pharmacy students towards their community pharmacy training experience: a cross-sectional study from Jordan

**DOI:** 10.1186/s12909-021-02596-w

**Published:** 2021-03-17

**Authors:** Rana Abu Farha, Eman Elayeh, Needa Zalloum, Tareq Mukattash, Eman Alefishat, Maysa Suyagh, Iman Basheti

**Affiliations:** 1 Department of Clinical Pharmacy and Therapeutics, Faculty of Pharmacy, Applied Science Private University, P.O. 11931, Amman, Jordan; 2 Department Biopharmaceutics and Clinical Pharmacy, Faculty of Pharmacy, The University of Jordan, Amman, Jordan; 3 Department Clinical Pharmacy, Faculty of Pharmacy, Jordan University of Science and Technology, Irbid, Jordan; 4 Department of Pharmacology, College of Medicine and Health Sciences, Khalifa University of Science and Technology, P O Box 127788, Abu Dhabi, United Arab Emirates; 5 Center for Biotechnology, Khalifa University of Science and Technology, Abu Dhabi, United Arab Emirates

**Keywords:** Training, Community pharmacy, Perception, Experience, Jordan

## Abstract

**Background:**

The fact that pharmacists are in the front line of patients’ care gives a great responsibility to focus on education and training of pharmacy students to build a ‘patient-centered’ clinicians. Unfortunately, pharmacy education in the developing countries, have been lagging behind actual practice delivered by pharmacists. This highlighted the need to evaluate the perceptions of undergraduate pharmacy students regarding their current pharmacy training practices and experiences.

**Methods:**

This is a cross-sectional study that was conducted in Jordan during the period from August 2018 to October 2018. During the study period, a questionnaire was distributed to pharmacy students to collect information regarding 1) pharmaceutical care services provided by them during their experiential training, 2) their perceptions towards training sites, 3) their perceptions of the outcomes of their training experience, 4) information about their training site and 5) their demographics characteristics.

**Results:**

A total of 202 pharmacy students responded to the questionnaire. The majority of them reported having the opportunity to dispense refill or new prescriptions (73.8%, *n* = 149), and conduct patient interviews (69.8%, *n* = 141, but they were not provided good opportunities to create electronic patient profiles using the information obtained (53.0%, *n* = 107), perform required dose calculations based on patient information (37.6%, *n* = 76), and interact with other healthcare professionals (34.6%, *n* = 70). In addition, students showed positive attitudes toward training sites, positive feedback about the outcomes of their training experience (median scores range between 4 and 5 for all statements (IQR = 1 for all)).

**Conclusion:**

Students showed positive feedback about the outcomes of their training experience, but they felt that the selected training sites do not have adequate resources to meet their training competencies. Memorandums of understanding development is needed to specify the purpose of training and define the responsibility for both parties of the training process.

**Supplementary Information:**

The online version contains supplementary material available at 10.1186/s12909-021-02596-w.

## Background

Pharmacists nowadays carry more responsibilities and commitments to improve their practice, emphasizing on the fact that the pharmacy profession is becoming more dynamic by the day [1, 2]. The role of pharmacists worldwide is expanding into a more patient-focused and outcome-oriented role compared to the previous dispensing role [1]. The establishment of evidence-based practice, including pharmaceutical care services, is essential [1, 3]. Pharmaceutical care is defined as “the responsible provision of drug therapy for the purpose of achieving definite outcomes that improve a patient’s quality of life” [4]. Pharmacists have been shown to have an optimistic attitude toward the implementation of new pharmaceutical care practices [5–7].

The adoption of pharmaceutical care by pharmacists all over the world is needed. Yet, several barriers have been identified, including lack of private counselling areas, lack of access to patient medical records, and most importantly lack of understanding of pharmaceutical care practices [8, 9]. A proposed solution to some of the barriers lie in establishing and modifying the undergraduate curriculum, including students’ training practices that is more focused on the optimal understanding of pharmaceutical care, providing needed knowledge and skills for students to be able to deliver different pharmaceutical care services successfully throughout their career [9, 10].

Unfortunately, a universal concern of a mismatch between actual pharmacy practice and pharmacy education has been acknowledged [8]. Pharmacy education and research in the developing countries, including the Middle East, have been lagging behind actual practice delivered by pharmacists [8]. Nevertheless, a substantial pace forward has been taken in the previous few years. This includes introducing changes into pharmacy education in academic institutions in many countries, including Jordan, which was driven in part by many of these institutions applying for international accreditation certification such as the Accreditation Council for Pharmacy Education (ACPE) standards. The main mission for ACPE standards is “the development of students who can contribute to the care of patients and to the profession by practicing with competence and confidence in collaboration with other health care providers. Also, the standards focus on the development of students’ professional knowledge, skills, attitudes, and values, as well as sound and reasoned judgment and the highest level of ethical behavior” [11].

To achieve this mission, pharmacy schools have in turn enhanced the training courses delivered to students to meet these learning objectives. Pharmacy students in all Jordanian universities are requested to complete a mandatory 1440 h of professional training in order for students to be equipped with the necessary pharmacy-related skills, including simple dispensing services, compounding extemporaneous preparations, providing medication counseling, implementing lengthy medication management review services, taking an appropriate medication histories, and responding to drug information inquiries [5, 12–14].

Assessment of learning competencies and outcomes achieved from this professional training is essential for quality improvement of pharmacy curriculum. Currently, no previous study has evaluated students’ perception towards their community pharmacy training experience. Thus, this study was designed to evaluate the perceptions of undergraduate pharmacy students regarding their current training practices and pinpoint the areas of weaknesses to improve them.

## Methods

### Study design and subjects

This is a cross-sectional study that was conducted in Jordan to investigate the perceptions of undergraduate pharmacy students regarding their current pharmacy training practices and experiences. To achieve this aim, a convenience sample of pharmacy students who are currently studying a bachelor’s degree in pharmacy or doctor of pharmacy from different accredited and non-accredited universities were recruited during the period from August 2018 to October 2018. Only students from the third academic year or higher who registered in their training in summer 2018 were considered to be eligible for participation since those at the first two years have not start their practice training yet.

### Sample size calculation

The standard formula: n = P × (1- P) × z^2^/d^2^ was used to calculate a minimal sample size where 96 students were considered a representative sample size for this study. We determined the sample size based on the most conservative proportion (*P* = 50%), and using 10% desired precision, and a confidence levels of 95%.

### Questionnaire development and data collection

Following an extensive review of the literature on studies conducted to assess students’ perceptions towards their pharmacy practice experience [15, 16], a questionnaire was designed in English language to address our intended aim of the study (**Supplementary material**). The initial draft of the questionnaire was evaluated by four independent academic experts to validate its content. All the experts had previous experience in pharmacy training and in questionnaire developments, and they provided their comments about the questionnaire items as track changes. Then, the primacy investigator of the study (RAF) incorporated the comments when appropriate to prepare the final version of the questionnaire. In the case of disagreement, the raised points were discussed until consensus was reached. At the end of this content validity, the questions were streamlined to ensure clarity and comprehensibility.

The questionnaire was then pilot tested on 10 students to provide their feedback on the comprehensibility and clarity of the questions. Data obtained from pilot testing was not included in the final analysis. The questionnaire’s internal consistency and reliability was assessed by measuring Cronbach’s α for each of the study domains, with values ≥0.922 for all, which indicates excellent internal consistency.

The questionnaire consisted of five primary domains: 1) assessment of pharmaceutical care services provided by students during experiential training, this part was assessed using yes or no choices. 2) Students’ perceptions towards training sites and, 3) students’ perceptions of the outcomes of their training experience. Perceptions of students were assessed using the 5-points Likert scale (strongly agree, agree, neutral, disagree, and strongly disagree). Also, the questionnaire included information regarding the 4) training site and 5) demographic characteristics of the students which included (gender, academic year, type of university, residential area, and academic major).

Data was collected by distributing the questionnaire using electronic platforms (Facebook and WhatsApp). The questionnaire was uploaded on google form, and the link was posted on pharmacy students’ Facebook groups for eight different Jordanian universities. Also, for some universities, students who registered in training courses, they provide their phone numbers for their academic tutors to follow them during their onsite training, so, the questionnaire link was distributed to them using WhatsApp platform. Only students who registered in training courses at the summer semester were asked to fill out the questionnaire. The first page of the questionnaire contained details about the study objective, voluntariness of participation, anonymity of the data collected, and the estimated time to complete the questionnaire. This was followed by an electronic consent where students were given the option of completing the questionnaire or terminating the study as follow “Clicking on the “agree“ button indicates that you have read the above information, and you voluntarily agree to participate, if you do not wish to participate in the research study, please decline participation by clicking on the “disagree” button”. The identity of all participants were kept anonymous by not requesting any personal identifying information, and data were kept at personal computer of the principle investigator using password protected files.

### Ethical consideration

The World Medical Association Declaration of Helsinki guidance was followed in the study [17]. The study was approved by the Institutional Review Board (IRB) committee at the Applied Science Private University (Approval No. 2019-PHA-6). Also, an electronic informed consent form was obtained from all participants (all of them aged > 18 years) before participation in the study.

### Statistical analysis

Following data collection, the questionnaire responses were coded and entered into a customized database using the Statistical Package for the Social Sciences (SPSS), Version 22.0 (IBM Corp., Armonk, New York, USA). Descriptive results were presented as median and interquartile range (IQR) for ordinal Likert scale variables, and percentages for nominal variables. Also, Cronbach’s α was used to evaluate the reliability of the questionnaire i.e. that the scales constructed are fit for its purpose, with values ≥0.7 indicates acceptable internal consistency [18].

## Results

### General and demographic characteristics of students

A total of 202 pharmacy students responded to the questionnaire. The majority of respondents were female (80.7%, *n* = 163), of BPharm degree (67.3%, *n* = 136), and from governmental universities (73.0%, *n* = 146). Also, most of them (86.6%, *n* = 175) were from ACPE accredited institutions. Results are summarized in Table 1.

**Table 1:** General information and demographic data of pharmacy students (*N* = 202)

Demographic data of pharmacy students	N (%*)
**University type**	
Private	54 (27.0)
Governmental	146 (73.0)
**From ACPE accredited Universities**	
Yes	175 (86.6)
No	27 (13.4)
**Gender**	
Male	39 (19.3)
Female	163 (80.7)
**Academic major**	
BSc pharmacy	136 (67.3)
Pharm D	66 (32.7)
**Year of study**	
3rd year	3 (1.5)
4th year	69 (34.2)
5th year	119 (58.9)
6th year	11 (5.4)
**Residential area**	
Amman	106 (52.5)
Others	96 (47.5)

*valid percent

### Training site general information

Around half of responding students (*n* = 106) were trained in Amman (the capital city of Jordan) with Irbid coming next (33.2%, *n* = 67). The majority of responding students practiced in independent pharmacies (68.8%, *n* = 121), and most practice sites had an average number of prescriptions of less than 50 per day (68.3%, *n* = 138). Of the practice sites, 57.4% (*n* = 116) had prescription software, and 64.9% (*n* = 131) had one or two active pharmacy tutors available at the site of training. The results are summarized in Table 2.

**Table 2:** Training site general information (*n* = 202)

Question	N (%)
**Community Pharmacy Practice site location**	
Amman	102 (50.5)
Irbid	67 (33.2)
Others	33 (16.3)
**Type of training site**	
Chain pharmacy	55 (31.3)
Independent pharmacy	121 (68.8)
**Average number of prescriptions dispensed per day at your practice site**	
Less than 50	138 (68.3)
50–99	40 (19.8)
100–149	19 (9.4)
150–199	0 (0)
200–249	3 (1.5)
250–299	0 (0)
More than 300	2 (1.0)
**Prescription software available**	
Yes	116 (57.4)
No	86 (42.6)
**Number of active pharmacy tutors available at the site at the time of your training**	
1	71 (35.1)
2	76 (37.6)
3	32 (15.8)
4 p	11 (5.4)
5 or more	12 (5.9)

### Pharmaceutical services provided by students during experiential training

The majority of students had the opportunity to dispense refill or new prescriptions (73.8%, *n* = 149), conduct patient interviews (69.8%, *n* = 141), respond to drug information inquiries (76.2%, *n* = 154), counsel patients on prescription medications (73.3%, *n* = 148) and Over the Counter (OTC) medications (73.3%, n = 148). Importantly, students were not provided good opportunities to create electronic patient profiles using the information obtained (53.0%, *n* = 107), perform required dose calculations based on patient information (37.6%, *n* = 76), interact with other healthcare professionals (e.g. physicians) (34.6%, *n* = 70), prepare and compound extemporaneous preparations (44.1%, *n* = 89), assess patient compliance to their treatment (34.7%, n = 70), and conduct physical assessments for patients when needed (39.1%, *n* = 79). Results are summarized in Table 3.

**Table 3:** Pharmaceutical services provided by pharmacy students during their experiential training experience (n = 202)

Pharmacy students’ opportunities	No	Yes
	N (%)	
1. Dispensing new/refill medication orders	53 (26.2)	149 (73.8)
2. Conducting patient interviews to obtain patient information	61 (30.2)	141 (69.8)
3. Creating electronic patient profiles using the information obtained	107 (53.0)	95 (47.0)
4. Responding to drug information inquiries	48 (23.8)	154 (76.2)
5. Interacting with other health care professionals	70 (34.7)	132 (65.3)
6. Counseling patients on prescription medications	54 (26.7)	148 (73.3)
7. Counseling consumers on OTC medications	54 (26.7)	148 (73.3)
8. Interpreting and evaluating patient information	63 (31.2)	139 (68.8)
9. Identifying patient-specific factors that affect health, pharmacotherapy, and/or disease state management	67 (33.2)	135 (66.8)
10. Performing required dose calculations based on patient information	76 (37.6)	126 (62.4)
11. Providing patient-centered care	67 (33.2)	135 (66.8)
12. Preparing and compounding extemporaneous preparations	89 (44.1)	113 (55.9)
13. Assessing patient compliance to their treatment	70 (34.7)	132 (65.3)
14. Conducting physical assessments for patients when needed	79 (39.1)	123 (60.9)
15. Interacting with pharmacy tutors in the delivery of pharmacy services	41 (20.3)	161 (79.7)

### Perception of students toward training site

Students showed positive attitudes toward their selected training sites with a median scores ranging between 4 and 5 for all statements (IQR = 1 for all). They believed that training sites were equipped with appropriate drug information resources (median = 4, IQR = 1). Also, they agreed that their training sites have adequate volume of OTC medications (median = 5, IQR = 1), and provide opportunities to ask the pharmacy tutors questions (median = 4, IQR = 1). The results are summarized in Table 4.

**Table 4:** Perception of students towards training sites (community pharmacies) (n = 202)

Statements	Median (IQR)
**General characteristics of the training sites**	
1. The training site where I practiced was equipped with appropriate drug information resources	4.0 (1.0)
2. The training site where I practiced was provided with a storage unit for all my belongings	4.0 (1.0)
3. The training site where I practiced appeared to have adequate volume of OTC medications	5.0 (1.0)
4. The training site where I practiced have a prescription volume adequate for effective learning during training	4.0 (1.0)
5. The training site where I practiced ensured patients’ confidentiality	4.5 (1.0)
6. The training site where I practiced had an adequate patient population needed to meet the learning objectives set for my training	5.0 (1.0)
7. The training site where I practiced provided pharmaceutical care services suitable for all people	4.0 (1.0)
8. The training site where I practiced provided a practice environment that supports students’ interaction with patients	4.0 (1.0)
9. The training site where I practiced provided opportunities to interact with other healthcare providers	4.0 (1.0)
10. The training site where I practiced provided opportunities to ask the pharmacy tutors questions	4.0 (1.0)
11. The training site where I practiced displayed a professional image (demonstrated ethical practice and evidence of patient-centered practice	4.5 (1.0)
12. The training site where I practiced was adequately staffed to provide quality pharmaceutical care services to patients	4.5 (1.0)

**IQR: interquartile range**

### Perception towards the outcomes of training experience

Students also revealed positive feedback about the outcomes of their training experience, where they reported that their training experience increased their involvement with pharmacy profession (median = 4, IQR = 1), and helped them to develop their social skills (median = 4, IQR = 1). The results are summarized in Table 5.

**Table 5:** Pharmacy Student perception about the outcomes of their training experience (n = 202)

Statements	Median (IQR)
1. The training experience increased my involvement with pharmacy profession	4.0 (1.0)
2. The training experience is an informal multi-cultural education	4.0 (1.0)
3. The training experience helped me to grow academically	4.0 (1.0)
4. The training experience helped me to develop social skills	4.0 (1.0)
5. The training experience helped me to increase my critical thinking skills	5.0 (1.0)
6. The training experience helped me to increase my self-esteem	4.0 (1.0)
7. The training experience is a development of lifelong learning skills / completion of experiential learning cycle	4.0 (1.0)

**IQR: interquartile range**

## Discussion

The fact that the pharmacists are in the ‘front line’ of patient care gives a great responsibility to focus on education and training of the pharmacists to have a leading role in patient care, supporting patients to make the most effective and cost-effective use of medications. And try to build a ‘patient-centered’ clinicians, with good interpersonal and communication skills. This highlighted the need for us to conduct this study. Our study was designed to evaluate the perceptions of pharmacy students in Jordan regarding their current pharmacy training practices provided by their schools of pharmacy and pinpoint the areas of weaknesses to improve them.

It has been known that the provision of pharmaceutical care should revolve around the ‘patient-care process’, where pharmacists use a patient-centered approach in collaboration with other health care providers to optimize medication use and health outcomes for the patients [19]. Accordingly, many practice modalities have been developed, from simple dispensing services to lengthy Medication Management Review services (MMR) applied in the outpatient and inpatient settings [5, 8, 13].

Interestingly, regarding the pharmaceutical care services provided by the participated students, our results showed that 76.2% of students were involved in responding to drug information inquiries, and 73.3% reported to counsel patients on prescription medications and OTC medications. Furthermore, 69.8% of them conducted patient interviews. Our suggestion is to give full responsibility for assigning students to specific training sites that ensure all professional and learning competencies are met. Also, a formal memorandum of understandings needs to be developed between both parties (the universities and the training sites) that will specify the purpose of training and define the responsibility and expectation of the practice regarding the education of the students.

Moreover, our findings suggest that students showed positive perception towards their selected training sites and positive feedback about the outcomes of their training experience. Different factors may contribute to these results, starting with the interaction between students and the site tutors. Around 64.9% of the students had one or two active pharmacy tutors available at the site of training , which increased the student opportunities to ask for the pharmacy tutors’ help to clarify different aspects of their training. Consequently, this may increase training efficiency and student satisfaction. Similar to our results, Zeitoun et al. reported higher rate of satisfaction in community pharmacy setting compared to the hospital setting, he attributed this to better interaction between students and site tutors at the community setting as the one to one ratio is higher and due to the nature of the workload in the hospital setting that lessens this interaction [15].

In addition to that, students reported to have an excellent opportunity to interact with patients. Around 70% of the students were able to conduct patient interviews to obtain patient information, this gave them a chance to practice how to apply communication skills, patients' counselling, and patients' interviews under the supervision of professional pharmacists, which will prepare them to be a ‘patient-centred’ member of the pharmacy team with good interpersonal and communication skills. Different studies support the importance of student-patient interaction. Interestingly, Erstad and Armstrong et al. found that students have a great desire to have increased communication with patients, as some students who spoke to patients frequently commented that they wished they had spoken to them more, this interaction has been reported to have a positive impact on the benefit of students' experience [20]. Furthermore, Zeitoun et al. found that students constructively tried to have increased interaction with patients. And that interaction was significant for students' satisfaction with their experience [15].

Although, this study is the first of its kind in Jordan and the Middle East, which gives it a great strength point, but it has several limitations. One obvious limitation is the analysis, where the comparisons between accredited and non-accredited universities were not feasible due to the sample size. Moreover, data was collected via a self-administered questionnaire which might have generated a risk of social desirable bias as students may not reflect their actual behaviors. Also, data was collected using online questionnaire which was distributed to specific social media groups, which could limit the generalizability of the results, also students accounts were not verified, and those students without Facebook accounts could not be able to reach the questionnaire link.

## Conclusion

First and foremost, this study serves as a baseline study that identified students’ perceptions towards their training experience, and towards the impact of proper training programs on  their training outcomes. Students showed positive perception towards their selected training sites and positive feedback about the outcomes of their training experience. Even though students met certain training competencies during their training which could help in maximizing patients’ health outcomes from their treatment regimens, but they were not given adequate opportunities to meet other competencies such as performing dose calculations, assessing patients’ adherence to medications and compounding extemporaneous preparations when needed. Memorandums of understanding development is needed to specify the purpose of training and define the responsibility for both parties of the training process.

## Supplementary Information

**Additional file 1.**

## Data Availability

The datasets used and/or analysed during the current study are available from the corresponding author on reasonable request.

## Abbreviations

ACPE: Accreditation Council for Pharmacy Education
IRB: Institutional Review Board
MMR: Medication Management Review services
OTC: Over the Counter
SPSS: Statistical Package for the Social Sciences

## References

[1] Al-Quteimat OM, Amer AM (2016). Evidence-based pharmaceutical care: the next chapter in pharmacy practice. Saudi Pharm J.

[2] Al-Qudah RA, Tuza O, Tawfiek H, Chaar B, Basheti IA. Community pharmacy ethical practice in Jordan: assessing attitude, needs and barriers. Pharm Pract (Granada) 2019;17(1):1386–1397, DOI: 10.18549/PharmPract.2019.1.1386. 10.18549/PharmPract.2019.1.1386 PMC6463417 31015876

[3] Al Alawneh M, Nuaimi N, Basheti IA. Pharmacists in humanitarian crisis settings: assessing the impact of pharmacist-delivered home medication management review service to Syrian refugees in Jordan. Res Social Adm Pharm 2019;15(2):164–172. doi: 10.1016/j.sapharm.2018.04.008. Epub Apr 10. 10.1016/j.sapharm.2018.04.008 29661563

[4] Hepler CDSLM (1990). Opportunities and responsibilities in pharmaceutical care. Am J Pharm Educ.

[5] Elayeh E, Akour A, Almadaeen S, AlQhewii T, Basheti IA (2017). Practice of pharmaceutical care in community pharmacies in Jordan. Trop J Pharm Res.

[6] Abu Hammour K, El-Dahiyat F, Abu FR (2017). Health care professionals knowledge and perception of pharmacovigilance in a tertiary care teaching hospital in Amman, Jordan. J Eval Clin Pract.

[7] Abu Hammour K, Abu Farha R, Basheti I (2016). Hospital pharmacy medication reconciliation practice in Jordan: perceptions and barriers. J Eval Clin Pract.

[8] Fathelrahman A, Izham M, Wertheimer A. Pharmacy practice in developing countries. The Pharm J. 2016;297(7892).

[9] Aburuz S, Al-Ghazawi M, Snyder A (2012). Pharmaceutical care in a community-based practice setting in Jordan: where are we now with our attitudes and perceived barriers?. The Int J Pharm Pract.

[10] Kheir N, Zaidan M, Younes H, El Hajj M, Wilbur K, Jewesson PJ (2008). Pharmacy education and practice in 13 Middle Eastern countries. Am J Pharm Educ.

[11] ACPE. Accreditation Council for Pharmacy Education. Accreditation Standards and Guidelines for the Professional Program in Pharmacy Leading to the Doctor of Pharmacy Degree. 2011.

[12] Ahmed Ibrahim Fathelrahman MIMI, Albert I, Wertheimer P. Pharmacy practice in developing countries. The Pharm J. 2016;297(7892).

[13] Basheti IA, Rizik M, Bulatova NR (2018). Home medication management review in outpatients with alarming health issues in Jordan: a randomized control trial. J Pharm Health Serv Res.

[14] Abu Farha R, Abu Hammour K, Mukattash T, Alqudah R, Aljanabi R (2019). Medication histories documentation at the community pharmacy setting: a study from Jordan. PLoS One.

[15] Zeitoun AA, El Zein HL, Zeineddine MM (2014). Effect of pharmacy practice program on pharmacy student learning, satisfaction, and efficiency: assessment of introductory pharmacy practice course. J Pharm Pract.

[16] Mulherin K, Ijaz N, Prata A, Cheng W (2012). Canadian Experiential Education Project for Pharmacy.

[17] World Medical A (2013). World medical association declaration of Helsinki: ethical principles for medical research involving human subjects. JAMA..

[18] Taber KS (2018). The use of Cronbach’s alpha when developing and reporting research instruments in science education. Res Sci Educ.

[19] JCI. Joint Commission of Pharmacy Practitioners. Pharmacists’ Patient Care Process. 2014.

[20] Erstad BL, Armstrong EP, Callahan P, Keller J (1997). Evaluation of practice site learning experience for entry-level doctor of pharmacy students. Am J Pharm Educ.

