# Supplementary material for: Perception of pharmacy students towards their community pharmacy training experience: a cross-sectional study from Jordan
Source: BMC Med Educ. 2021 Mar 17;21:161. doi: 10.1186/s12909-021-02596-w (PMC7967969; doi:10.1186/s12909-021-02596-w)
Supplement: Supplementary file 1 — Additional file 1. [file 12909_2021_2596_MOESM1_ESM.docx]

**Perception of pharmacy students towards their community pharmacy training experience: a cross-sectional study from Jordan**

Running title: Students’ perception to pharmacy training

**Rana Abu Farha^1^, Eman Elayeh^2^, Needa Zalloum^2^, Tareq Mukattash^3^, Eman Alefishat^2,4,5*^, Maysa Suyagh^2^, Iman Basheti^1^**

1. Department of Clinical Pharmacy and Therapeutics, Faculty of Pharmacy, Applied Science Private University, Amman-Jordan. P.O. 11931
2. Department Biopharmaceutics and Clinical Pharmacy, Faculty of Pharmacy, The University of Jordan, Amman, Jordan.
3. Department Clinical Pharmacy, Faculty of Pharmacy, Jordan University of Science and Technology, Irbid, Jordan.
4. Department of Pharmacology, College of Medicine and Health Science, Khalifa University of Science and Technology, Abu Dhabi, United Arab Emirates
5. Center for Biotechnology, Khalifa University of Science and Technology, Abu Dhabi, United Arab Emirates

***Corresponding and senior author**

Eman Alefishat

Department of Pharmacology, College of Medicine and Health Science, Khalifa University of Science and Technology, Abu Dhabi, United Arab Emirates. Email: [Eman.alefishat@ku.ac.ae](mailto:Eman.alefishat@ku.ac.ae); Tel: +9715018466 P O Box 127788, Abu Dhabi, UAE

**Evaluation of Community Pharmacy Training Experience as Perceived by Jordanian Pharmacy Students a cross-sectional study**

**Dear Students**

Researchers from different Jordanian Universities are carrying out a research project to assess the perceptions of pharmacy students towards their community pharmacy training experience.

This survey was developed for this purpose; it may take you up to 15 minutes to be filled.

We would like to confirm that all information provided here will be kept confidential, and will be used only for research purposes.

**ELECTRONIC CONSENT**: Please select your choice below.

Clicking on the "agree" button below indicates that:

1. You have read the above information
2. You voluntarily agree to participate

If you do not wish to participate in the research study, please decline participation by clicking on the "disagree" button.

- Agree
- Disagree

Your participation in completing this survey is highly appreciated.

Note: In this questionnaire the term "pharmacy preceptor" refers to the community pharmacist working at the training site.

**Part 1. General information and demographic data of pharmacy student**

**University type**

- Private
- Governmental

**From ACPE accredited Universities**

- Yes
- No

**Gender**

- Male
- Female

**Academic major**

- BSc pharmacy
- Pharm D

**Year of study**

- 3rd year
- 4th year
- 5th year
- 6th year

**Residential area**

- Amman
- Others

**Part 2: Training site general information**

**Community Pharmacy Practice site location**

- Amman
- Irbid
- Others

**Type of training site**

- Chain pharmacy
- Independent pharmacy

**Average number of prescriptions dispensed per day at your practice site**

- Less than 50
- 50-99
- 100-149
- 150-199
- 200-249
- 250-299
- More than 300

**Prescription software available**

- Yes
- No

**Number of active pharmacy preceptors available at the site at the time of your training**

- 1 pharmacist
- 2 pharmacists
- 3 pharmacists
- 4 pharmacists
- 5 pharmacists or more

**Part 3: Pharmaceutical services provided by students during experiential training**

**Which of the following pharmaceutical services you allowed to provide at your training site?**

| **Pharmacy students’ opportunities** | No | Yes |
| --- | --- | --- |
|  |  | |
| 1. Dispensing new/refill medication orders |  |  |
| 1. Conducting patient interviews to obtain patient information |  |  |
| 1. Creating electronic patient profiles using the information obtained |  |  |
| 1. Responding to drug information inquiries |  |  |
| 1. Interacting with other health care professionals |  |  |
| 1. Counseling patients on prescription medications |  |  |
| 1. Counseling consumers on OTC medications |  |  |
| 1. Interpreting and evaluating patient information |  |  |
| 1. Identifying patient-specific factors that affect health, pharmacotherapy, and/or disease state management |  |  |
| 1. Performing required dose calculations based on patient information |  |  |
| 1. Providing patient-centered care |  |  |
| 1. Preparing and compounding extemporaneous preparations |  |  |
| 1. Assessing patient compliance to their treatment |  |  |
| 1. Conducting physical assessments for patients when needed |  |  |
| 1. Interacting with pharmacy preceptors in the delivery of pharmacy services |  |  |

**Part 4: Perception of students towards training sites (community pharmacies)**

Please rate your level of agreement with the following statements

| **Statements** | **Strongly agree** | **Agree** | **Neutral** | **Disagree** | **Strongly disagree** |
| --- | --- | --- | --- | --- | --- |
| 1. The training site where I practiced was equipped with appropriate drug information resources |  |  |  |  |  |
| 1. The training site where I practiced was provided with a storage unit for all your belongings |  |  |  |  |  |
| 1. The training site where I practiced appear to have adequate volume of OTC medications |  |  |  |  |  |
| 1. The training site where I practiced have a prescription volume adequate for effective learning during training |  |  |  |  |  |
| 1. The training site where I practiced ensured patients' confidentiality |  |  |  |  |  |
| 1. The training site where I practiced had an adequate patient population needed to meet the learning objectives set for my training |  |  |  |  |  |
| 1. The training site where I practiced provided pharmaceutical care services suitable for all people |  |  |  |  |  |
| 1. The training site where I practiced provided a practice environment that supports students' interaction with patients |  |  |  |  |  |
| 1. The training site where I practiced provided opportunities to interact with other healthcare providers |  |  |  |  |  |
| 1. The training site where I practiced provided opportunities to ask the pharmacy preceptors questions |  |  |  |  |  |
| 1. The training site where I practiced displayed a professional image (demonstrated ethical practice and evidence of patient-centered practice |  |  |  |  |  |
| 1. The training site where I practiced was adequately staffed to provide quality pharmaceutical care services to patients |  |  |  |  |  |

**Part 5: Perception towards the outcomes of training experience**

Please rate your level of agreement with the following statements

| **Statements** | **Strongly agree** | **Agree** | **Neutral** | **Disagree** | **Strongly disagree** |
| --- | --- | --- | --- | --- | --- |
| 1. The training experience increased your involvement with pharmacy profession |  |  |  |  |  |
| 1. The training experience is informal multi-cultural education |  |  |  |  |  |
| 1. The training experience helped you to grow academically |  |  |  |  |  |
| 1. The training experience helped you to develop social skills |  |  |  |  |  |
| 1. The training experience helped you to increase your critical thinking skills |  |  |  |  |  |
| 1. The training experience helped you to increase self-esteem |  |  |  |  |  |
| 1. The training experience is a development of lifelong learning skills / completion of experiential learning cycle |  |  |  |  |  |
